# Joint Effects of Intraocular Pressure and Myopia on Risk of Primary Open-Angle Glaucoma: The Singapore Epidemiology of Eye Diseases Study

**DOI:** 10.1038/srep19320

**Published:** 2016-01-13

**Authors:** Yih-Chung Tham, Tin Aung, Qiao Fan, Seang-Mei Saw, Rosalynn Grace Siantar, Tien Y. Wong, Ching-Yu Cheng

**Affiliations:** 1 Singapore Eye Research Institute, Singapore National Eye Centre, Singapore; 2 Department of Ophthalmology, Yong Loo Lin School of Medicine, National University of Singapore and National University Health System, Singapore; 3 National Healthcare Group Eye Institute, Tan Tock Seng Hospital, Singapore; 4 Duke-NUS Medical School, Singapore

## Abstract

We examined the joint effects of intraocular pressure (IOP) and myopia on the risk of primary open angle glaucoma (POAG) in a multi-ethnic Asian population. A total of 9,422 participants (18,469 eyes) in the Singapore Epidemiology of Eye Diseases Study were included. Of them, 213 subjects (273 eyes) had POAG. All participants underwent standardised examinations. The independent and joint effects of IOP and myopia on POAG were examined using logistic regression models. Generalised estimating equation models were used to account for correlation between eyes. Higher IOP, longer axial length, and more negative spherical equivalent were independently associated with POAG, after adjusting for relevant covariates (all P ≤ 0.005). Significant interaction between IOP and myopia on POAG was observed (*P* interaction = 0.025). Eyes with moderate-to-high myopia (<−3.0 dioptres) with high IOP (≥20 mmHg) were 4.27 times (95% CI, 2.10–8.69) likely to have POAG, compared to eyes without myopia (>−0.5 dioptres) and with IOP <20 mmHg. Eyes with AL of ≥25.5 mm and high IOP (≥20 mmHg) were 16.22 times (95% CI, 7.73 to 34.03) likely to have POAG, compared to eyes with shorter AL (<23.5 mm) and lower IOP (<20 mmHg). These findings may provide additional insights into the pathophysiology of POAG and are particularly relevant for Asian populations.

Glaucoma is a leading cause of global irreversible blindness. Primary open angle glaucoma (POAG) is the most common form of glaucoma and affects 44.1 million individuals worldwide^1^. Furthermore, there is a disproportionate burden of POAG in Asia, with Asians accounting for 53% (23.5 million) of POAG cases worldwide^1^,^2^. While early detection of POAG is important in delaying or halting the progression of the disease^3^,^4^, a better understanding of the interplay between major risk factors for POAG is crucial^3^.

Intraocular pressure (IOP) is an established risk factor for POAG^3^,^5^, and is the only modifiable risk factor for the development and progression of POAG^6^,^7^. It has been postulated that elevated IOP exerts mechanical stress on the optic nerve head (ONH) and lamina cribrosa, and its adjacent tissues^4^,^8^. In addition, IOP-induced strain may also compress the lamina cribrosa and disrupt axonal transport of trophic factors which are essential to the autoregulation and survival of retinal ganglion cells^4^,^8^,^9^. As lamina cribrosa is the site where retinal ganglion cell axons congregate before traversing to the brain^10^, excessive mechanical strain at this structure may initiate glaucomatous damage^4^,^11^.

Many previous studies have also reported myopia as another important risk factor for POAG^12^,^13^,^14^,^15^. This association is stronger in individuals with moderate-to-high myopia (<−3.00 dioptre)^12^,^13^,^16^,^17^,^18^. This may reflect longer axial length (AL) in eyes with myopia which may be related to weaker connective tissue support at the ONH and lamina cribrosa region^13^,^14^. Eyes with myopia may thus have greater susceptibility for lamina cribrosa deformation which may lead to subsequent development of POAG^10^,^14^.

Although IOP and myopia are both closely linked to mechanical strain and deformation of the lamina cribrosa, the inter-relationship between IOP and myopia on the risk of POAG has not been well studied. Most previous studies have evaluated the independent effects of IOP and myopia on POAG, but have not explored the joint effects. This issue is especially important and relevant in East Asians, considering the increasing burden of myopia in this region, and the burden of POAG among Asians compared to other populations^1^,^19^.

The aim of this study is to assess the joint effects of IOP and myopia on POAG in a multi-ethnic Asian population. Findings in this study may provide greater insights into the pathophysiology of POAG, and are relevant to the increasing number of patients with myopia, particularly in Asia. Furthermore, joint effect analysis may also provide new knowledge to better stratify and identify individuals who are at higher risk of having POAG.

## Methods

### Study Populations

The Singapore Epidemiology Eye Disease (SEED) Study is a population-based cross sectional study, comprising of three major ethnic groups in Singapore: Malays (the Singapore Malay Eye Study, year 2004 to 2006), Indians (the Singapore Indian Eye Study, year 2007 to 2009), and Chinese (the Singapore Chinese Eye Study, year 2009 to 2011). Details of the study design and methodology of the SEED study have been reported elsewhere^20^,^21^,^22^. In brief, the study was conducted in the southwestern part of Singapore, using a standardized study protocol across the 3 ethnic groups of subjects. Age-stratified random sampling strategy was adopted in each ethnic group to select adults aged 40 to 80 years. Overall, a total of 4,168 Malays, 4,497 Indians, and 4,605 Chinese were identified and invited to participate in the study. Of which, 10,033 subjects participated and underwent the study examinations, including 3,280 Malays (78.7% response rate), 3,400 Indians (75.6% response rate), and 3,353 Chinese (72.8%). The study was approved by the Singapore Eye Research Institute Institutional Review Board. All participants gave a written informed consent and the conduct of the study adhered to the Declaration of Helsinki.

### Clinical Ocular Examinations

All subjects underwent a standardized interview, and ocular examinations at the Singapore Eye Research Institute^20^,^21^,^22^. In brief, intraocular pressure (IOP) was measured using the Goldmann applanation tonometer (GAT, Haag-Streit, Bern, Switzerland) before pupil dilation. One reading was taken from each eye. If the IOP reading was greater than 21 mm Hg, a repeat reading was taken, and the second reading was used for analysis. Central corneal thickness (CCT) was measured using an ultrasound pachymeter (Advent; Mentor O & O Inc., Norwell, Massachusetts, USA); the mean of 5 measurements was used for analysis. Refractive error was measured using an autorefractor (Canon RK-5 Autorefractor Keratometer; Canon Inc., Tokyo, Japan); the mean of the 5 measurements was used for analysis. Spherical equivalent (SE) was calculated as the spherical value plus half of the negative cylinder value. AL was measured using non-contact partial coherence interferometry (IOL Master V3.01, Carl Zeiss Meditec AG, Jena, Germany); the means of the 5 measurements were used for analysis. Gonioscopy was performed with a Goldmann two-mirror lens (Ocular Instruments, Inc., Bellevue,WA) under standard dark illumination in three groups of participants: glaucoma suspects (definition as below), all participants with temporal peripheral Van Herick grade 2 or less, and one in five randomly selected participants who did not meet the first two criteria.

After pupil dilation, the optic disc was evaluated using a +78D lens at 10 times magnification with a measuring graticule (Haag-Streit) during slit lamp funduscopy (Haag-Streit model BQ-900; Haag-Streit, Switzerland). The clinical vertical cup-to-disc ratio (VCDR) was calculated accordingly and morphological features such as disc haemorrhage, notching of the neural retinal rim, and retinal nerve fiber layer defects were documented.

### Visual Field Examinations

Static automated perimetry (Swedish Interactive Threshold Algorithm standard 24-2, Humphrey Field Analyzer II; Carl Zeiss Meditec, Dublin, CA) was performed on one in five participants and in all glaucoma suspects (definition as below). A visual field was defined as reliable when fixation losses were less than 20%, and false-positive, false-negative rates were less than 33%. Visual field test was repeated if the test result was unreliable. A glaucomatous visual field defect was defined as the presence of three or more significant (p < 0.05) non-edge contiguous points with at least one at the p < 0.01 level on the same side of the horizontal meridian in the pattern deviation plot, and classified as “outside normal limits” on the Glaucoma Hemifield Test, confirmed on 2 consecutive visual field examinations.

### Other Measurements

A detailed interviewer-administered questionnaire was used to collect demographic data, current and past medication history, and ocular surgery history.

### Glaucoma Diagnostic Definitions

Glaucoma suspect was defined as having any of the following criteria: (1) IOP > 21 mm Hg, (2) VCDR >0.6 or VCDR asymmetry >0.2 (3) signs consistent with pseudoexfoliation or pigment dispersion syndrome, (4) narrow angles (posterior trabecular meshwork visible for <180 degrees during static gonioscopy), and (5) peripheral anterior synechiae, (6) other findings consistent with secondary glaucoma, and (7) known history of glaucoma.

Glaucoma was defined according to the International Society of Geographical and Epidemiological Ophthalmology (ISGEO) criteria based on three categories^23^. Category 1 cases were defined as optic disc abnormality (VCDR/VCDR asymmetry ≥97.5 percentile or neuroretinal rim width between 11 and 1 o’clock or 5 and 7 o’clock <0.1 VCDR), with a corresponding glaucomatous visual field defect. Category 2 cases were defined as having a severely damaged optic disc (VCDR or VCDR asymmetry ≥99.5th percentile) in the absence of adequate performance in a visual field test. Category 3 cases were defined as subjects without visual field or optic disc data who were blind (corrected visual acuity, <3/60) and who had previous glaucoma surgery or had IOP >99.5 percentile.

A narrow anterior chamber angle was diagnosed if the posterior trabecular meshwork was seen for 180° or less of the angle circumference during static gonioscopy. Primary angle-closure glaucoma (PACG) was defined as an eye with glaucoma accompanied with the presence of narrow anterior chamber angle and features of trabecular obstruction by peripheral iris (such as peripheral anterior synechiae, elevated IOP, iris whirling, ‘glaukomflecken’ lens opacities, or excessive pigment deposition on the trabecular surface). Subjects with glaucoma and an open, normal drainage angle with no identifiable secondary pathologic processes were defined as having POAG. In this study, PACG and secondary glaucoma cases (i.e. pseudoexfoliative glaucoma, neovascular glaucoma, traumatic glaucoma) were excluded from the analysis.

### Statistical Analysis

We used eye-specific data and generalized estimating equation (GEE) models with exchangeable correlation structures were applied to account for the correlation between pairs of eyes for each individual.

IOP, myopia status and AL were analyzed as continuous and also as categorical exposure variables. IOP level was categorized into groups of ≥20 mmHg and <20 mmHg. We adopted a more conservative cutoff of 20 mmHg instead of the conventional 21 mmHg as previous Asian population-based studies reported a slightly lower upper-normative limit (97.5 percentile) for IOP^22^,^24^,^25^,^26^. Refractive myopia was categorised as non-myopia (SE: >−0.50D), low to moderate myopia (SE: −0.5D to −3.0D), and moderate-to-high myopia (SE: <−3.0D) in phakic eyes. Eyes with history of cataract surgery were removed for refractive myopia-related analysis. On the other hand, severity of axial myopia was categorised into 4 categories according to previous literature^27^,^28^, namely, AL of <23.5 mm, AL of 23.5 mm to <24.5 mm, AL of 24.5 mm to <25.5 mm, and AL of ≥25.5 mm. Multiple logistic regression with GEE were used to assess the independent and joint effects of IOP and myopia/ AL on POAG, while adjusting for age, gender, ethnicity and CCT. Statistical interactions between IOP and myopia status, and between IOP and AL categories were examined in separate models by including cross-product interaction terms in the corresponding logistic regression models. All statistical analyses were performed using Stata 12.1 (StataCorp LP, College Station, TX).

## Results

Of the 10,033 study subjects, 611 subjects had incomplete or missing measurements of clinical VCDR, IOP, AL or refraction, leaving a total of 9,422 subjects (18,469 eyes) for this analysis.

Among the included participants, POAG was present in 213 participants (273 eyes). Overall, participants who had POAG were older, and more likely to be male and Malay (Table 1). In addition, participants who had POAG had significantly higher IOP, longer AL, more negative spherical equivalent refraction, thinner CCT, and larger clinical VCDR (all P ≤ 0.002) (Table 2). Of the 273 POAG eyes, 215 eyes had IOP <20 mmHg.

Table 3 shows the associations between ocular factors and POAG after adjusting for age, gender, ethnicity, IOP, CCT and AL. Overall, higher IOP (per mmHg increase; OR, 1.16; 95% CI 1.11 to 1.21), longer AL (per mm increase; OR, 1.26; 95% CI 1.17 to 1.36), and more negative SE (per negative dioptre; OR, 1.07; 95% CI 1.02 to 1.12), were associated with POAG. Further analyses showed that eyes with IOP ≥20 mmHg were 3.26 times (95% CI, 2.38 to 4.47) likely to have POAG, compared to eyes with IOP <20 mmHg. Eyes with AL ≥25.5 mm were 3.30 times (95% CI, 2.04 to 5.33) likely to have POAG, compared to eyes with AL <23.5 mm. In addition, when compared to eyes without myopia, moderate-to-high myope eyes were significantly associated with POAG (OR, 2.11; 95% CI, 1.44 to 3.09).

We further assessed the combined effects of IOP and refractive myopia, while adjusted for age, gender, ethnicity and CCT. Significant interaction was observed between IOP and refractive myopia on POAG (*P* for interaction = 0.025) (Table 4). Compared to eyes without myopia and with IOP <20 mmHg, low myopia eyes with IOP ≥20 mmHg were 4.52 times (95% CI, 2.66 to 7.68) likely to have POAG, and moderate-to-high myopia eyes with IOP ≥20 mmHg were 4.27 times (95% CI, 2.10 to 8.69) likely to have POAG. Consistently, we also observed strong joint effects between IOP and axial myopia on POAG. Eyes with AL of ≥25.5 mm and high IOP (≥ 20 mmHg) were 16.22 times (95% CI, 7.73 to 34.03) likely to have POAG, compared to eyes with shorter AL (<23.5 mm) and lower IOP (<20 mmHg) (Table 5). Nevertheless, the interaction between IOP and axial myopia on POAG was of borderline significance (*P* for interaction = 0.066).

## Discussion

We evaluated the joint effects of IOP and myopia on the risk of POAG in this study of nearly 10,000 Asian participants. To the best of our knowledge, this is the first population study which demonstrated a significant interaction and effect modification between IOP and myopia (both refractive and axial) on the risk of POAG. Our study substantiates the concept of biological interaction between IOP and myopia on the development of POAG, and is relevant to many patients with moderate and high myopia around the world, particularly so in Asia.

We found that higher IOP and longer AL, were independently associated with increased risk of POAG. These findings were consistent with that reported by previous cross-sectional and prospective population-based studies in Asians, Caucasians, African descents and Hispanics^16^,^18^,^29^,^30^,^31^,^32^,^33^. In a separate analysis which excluded eyes with history of cataract surgery, we observed that eyes with moderate-to-high myopia was associated with POAG but not eyes with low myopia. This was also similarly shown in previous studies^12^,^13^,^16^,^17^,^18^, further corroborating that higher myopia was associated with POAG.

More importantly, we observed strong combined effects between high IOP and myopia on POAG, which were greater than the expected additive effects of both exposures, thus suggestive of the presence of synergistic effect. Eyes with high IOP and moderate-to-high myopia had approximately 4.5-fold increased risk of POAG, compared to eyes without myopia and with relatively lower IOP. This trend was similarly shown in the joint effect analysis of IOP and AL, where eyes with high IOP and AL of ≥25.5 mm had approximately 16-fold increased risk of POAG. Importantly, we observed significant interaction between IOP and refractive myopia on POAG. Furthermore, our additional sensitivity analysis further showed that the trend of joint associations between IOP and refractive myopia on POAG remained largely similar after further adjusting for IOP lowering treatment (i.e. medication or surgery). Interaction between IOP and refractive myopia on POAG was still significant even after adjusting for IOP lowering treatment (*P* for interaction = 0.029, data not shown). Nevertheless, despite the strong effect observed in eyes with high IOP and long AL, the interaction between IOP and AL on POAG was of borderline significance. This may be due to the limited number of POAG cases which had both high IOP and long AL. Thus, future evaluation with greater statistical power is required to further ascertain the joint effect of IOP and AL on POAG. On the other hand, the greater effect estimate observed in eyes with high IOP and longer AL may indicate that evaluation of AL together with IOP measurement may potentially be more informative in stratifying individuals who are at higher risk of having POAG, compared to evaluation of refractive myopia status.

The observed significant interaction between IOP and refractive myopia on POAG suggests that IOP and myopia may act synergistically on the development of POAG, potentially via interdependent biomechanical processes. This further confirms the postulation that increased glaucoma susceptibility in myopic eyes may be further aggravated with the presence of elevated IOP, and vice versa^12^. One possible explanation is that high myopia eyes with increased AL may be associated with greater scleral thinning, poorer elasticity of the sclera wall and thus are more vulnerable to scleral tension across the ONH and lamina cribrosa region^34^,^35^. The concurrence of raised IOP in high myopia eyes is likely to further induce mechanical strain on an inherently ‘vulnerable’ ONH and lamina region, thus more readily leading to compression of retinal ganglion cell axons, followed by axonal damage and degeneration.

Similarly, in a 4-year prospective population-based study in Hispanic populations, Jiang *et al.* also observed that the risk of developing POAG was higher in those with higher baseline IOP and longer baseline AL. However, they did not observe significant interaction between baseline IOP and AL on incident POAG^29^. The nil observation from Jiang et al. may be due to limited statistical power as that study only comprised of 87 incident POAG cases and only one study eye in each subject was selected for the final analysis. On the other hand, Asians have higher prevalence of myopia, in particular high myopia, compared to other ethnicities^19^,^27^,^36^, thus it is possible that the multiplicative effect between IOP and myopia on POAG is more prominently observed in Asians. Nevertheless, future prospective population-based studies in Asians are still required to validate our current findings.

In this study, high IOP was observed to compound the risk of POAG in myopic eyes (and vice versa), above the risk estimates conferred by either factor alone. This information may potentially aid in the formulation of more targeted screening strategies for high-risk sub groups. In particular, individuals identified with concurrence of high IOP and high myopia should be warranted for more regular glaucoma screenings in order to detect glaucoma at an earlier stage. In addition, our joint effect finding may also help to guide treatment decisions for ocular hypertensive patients. Specifically, ocular hypertensive patients presenting with high myopia may be indicated for early IOP-lowering prophylactic treatment as compared to ocular hypertensives without myopia.

The strengths of our study include large population datasets across the 3 main ethnicities in Asia and utilisation of data from both eyes of each subject by adopting GEE modelling approach. This approach allowed inclusion of greater number of POAG cases and thus greater statistical power especially for stratified analyses. However, this study also has limitations. There might be a small proportion of POAG misclassifications in eyes of high myopia with large discs or tilted discs, in which accurate judgement of the ONH appearance is known to be challenging^24^,^35^. Such misclassifications were likely for cases which were defined based on ONH appearance alone (i.e. ISGEO criteria category 2). Nevertheless, in a sub-analysis which only included POAG cases defined based on both ONH appearance and corresponding visual field defects (i.e. ISGEO criteria category 1), we observed similar trends of associations between IOP, myopia and POAG, albeit slightly larger *P* values (data not shown). Furthermore, in our analyses, we did not further sub-stratify refractive myopia status into high myopia (defined as <−5.0D) due to limited POAG cases which had both high refractive myopia and high IOP (≥20 mmHg) in our sample. Hence, the trend of association in the high refractive myopia group was not evaluated. Future work with larger sample in high refractive myopia group is warranted to further validate our findings.

In conclusion, we found that high IOP and myopia had a synergistic effect on the risk of POAG in a population-based sample of nearly 10,000 Asian participants. Our findings provide additional insights into the pathophysiology of POAG, and may also help in the formulation of more effective, targeted glaucoma screening strategies in patients with myopia.

## Additional Information

**How to cite this article**: Tham, Y.-C. *et al.* Joint Effects of Intraocular Pressure and Myopia on Risk of Primary Open-Angle Glaucoma: The Singapore Epidemiology of Eye Diseases Study. *Sci. Rep.* **6**, 19320; doi: 10.1038/srep19320 (2016).

## Figures and Tables

**Table 1:** Demographic Characteristics of Study Population.

	Persons without Glaucoma (N = 9,209)	Persons with POAG (N = 213)	P value*
Age, year	58.2 (10.1)	63.4 (10.8)	<0.001
Male sex, n (%)	4,509 (49.0%)	130 (61.0%)	<0.001
Ethnicity, n (%):			
Malay	2,739 (29.7%)	110 (51.6%)	<0.001
Indian	3,275 (35.6%)	46 (21.6%)	
Chinese	3,195 (34.7%)	57 (26.7%)	

POAG = primary open-angle glaucoma. Data presented as means (SD) or number (%) as appropriate. *N*, number of subjects.

*Comparison between normal and POAG subjects by unpaired *t*-tests for age, and chi-square tests for gender and ethnicity.

**Table 2:** Clinical Characteristics of Study Eyes.

	Non-glaucoma eyes (n = 18,196)	POAG eyes (n = 273)	P value*
Intraocular pressure, mmHg	15.1 (3.2)	16.7 (4.7)	<0.001
Axial length, mm	23.6 (1.3)	24.0 (1.3)	<0.001
Spherical equivalent, D†	−0.30 (2.37)	−0.75 (2.88)	0.002
Central corneal thickness, μm	544.5 (34.1)	536.9 (32.9)	<0.001
Vertical cup-to-disc ratio	0.40 (0.12)	0.73 (0.13)	<0.001

Data are presented as mean (standard deviation) and are eye-specific.

†Excluding eyes with history of cataract surgery.

*Comparison between normal and glaucoma eyes using logistic regression models with generalized estimating equations.

**Table 3:** Association between Ocular factors and Primary Open-Angle Glaucoma.

Ocular Factors	POAG, n = 273			
	Model 1*		Model 2**	
	OR (95% CI)	P value	OR (95% CI)	P value
IOP:				
Per mmHg increase	1.15 (1.10 to 1.20)	<0.001	1.16 (1.11 to 1.21)	<0.001
≥ 20 mmHg (vs <20 mmHg)	3.13 (2.31 to 4.24)	<0.001	3.26 (2.38 to 4.47)	<0.001
Axial length (AL):				
Per mm increase	1.22 (1.13 to 1.31)	<0.001	1.26 (1.17 to 1.36)	<0.001
AL <23.5 mm	1.00 (reference)	-	1.00 (reference)	-
23.5 mm ≤ AL <24.5 mm	1.63 (1.18 to 2.26)	0.003	1.68 (1.20 to 2.34)	0.002
24.5 mm ≤ AL <25.5 mm	2.05 (1.35 to 3.13)	0.001	1.98 (1.30 to 3.03)	<0.001
AL ≥ 25.5 mm	3.14 (1.94 to 5.06)	<0.001	3.30 (2.04 to 5.33)	<0.001
Refractive status (SE):†				
Per negative dioptre	1.07 (1.02 to 1.12)	0.003	1.07 (1.02 to 1.12)	0.004
Non-myope (>−0.50D)	1.00 (reference)	-	1.00 (reference)	-
Low-myope (−0.5D to −3.0D)	1.03 (0.77 to 1.38)	0.834	1.08 (0.78 to 1.51)	0.638
Moderate-to-high myope (<−3.00D)	2.36 (1.65 to 3.37)	<0.001	2.11 (1.44 to 3.09)	<0.001

POAG = Primary Open-Angle Glaucoma; IOP = Intraocular pressure; SE = Spherical Equivalent; CCT = Central corneal thickness; OR = Odds ratio; CI = Confidence interval.

*Adjusted for age, gender, ethnicity.

**Adjusted for age, gender, ethnicity, IOP, CCT and axial length.

^†^Eyes with history of cataract surgery were excluded. Axial length was removed in this model because of collinearity with spherical equivalent.

**Table 4:** Stratum Specific-Odds Ratio (95% Confidence Interval) of POAG in Categorical Groups of IOP and Myopia Status†.

	Myopia Status‡								
	Non-Myope (>−0.50D)			Low Myope (−0.50D to −3D)			Moderate-to-High Myope (<−3.0D)		
**IOP status**	**n**	**(% of cases)**	**OR (95% CI)**	**n**	**(% of cases)**	**OR (95% CI)**	**n**	**(% of cases)**	**OR (95% CI)**
< 20 mmHg	11,352	1.2%	1.00 (reference)	3,011	1.0%	0.87 (0.58 to 1.31)	1,593	2.0%	2.28 (1.49 to 3.49)**
									
≥ 20 mmHg	1,136	3.1%	2.62 (1.73 to 4.00)**	378	6.3%	4.52 (2.66 to 7.68)**	185	5.4%	4.27 (2.10 to 8.69)**

POAG = primary open angle glaucoma; IOP = Intraocular pressure; OR = odds ratio.

†Analysis adjusted for age, gender, ethnicity and central corneal thickness.

‡ Eyes with history of cataract surgery were excluded.

**Denotes P value <0.001.

P interaction = 0.025

**Table 5:** Stratum-Specific Odds Ratio (95% Confidence Interval) of POAG in Categorical Groups of IOP and Axial Length†.

IOP status	Axial Length (AL) Status											
	AL < 23.5 mm			23.5 mm ≤ AL < 24.5 mm			24.5 mm ≤ AL < 25.5 mm			AL ≥ 25.5 mm		
	n	(% of cases)	OR (95% CI)	n	(% of cases)	OR (95% CI)	n	(% of cases)	OR (95% CI)	n	(% of cases)	OR (95% CI)
<20 mmHg	8,650	0.8%	1.00 (reference)	5,058	1.6%	1.76 (1.22 to 2.54)*	1,795	1.1%	1.57 (0.92 to 2.69)	1,212	2.0%	2.85 (1.60 to 5.08)**
												
≥20 mmHg	948	2.5%	2.97 (1.80 to 4.90)**	506	3.6%	3.93 (2.23 to 6.90)**	190	9.5%	9.74 (5.17 to 18.36)**	110	10.0%	16.22 (7.73 to 34.03)**

POAG = primary open angle glaucoma; IOP = intraocular pressure; OR = odds ratio.

†Analysis adjusted for age, gender, ethnicity and central corneal thickness.

*Denotes P value of <0.05.

**Denotes P value of <0.001.

P interaction = 0.066
